# Prevalence of airflow obstruction in patients with stable systolic heart failure

**DOI:** 10.1186/s12890-016-0351-9

**Published:** 2017-01-06

**Authors:** Morten Dalsgaard, Louis Lind Plesner, Morten Schou, Erik Kjøller, Jørgen Vestbo, Kasper Iversen

**Affiliations:** 1 Department of Cardiology, Herlev Hospital, Copenhagen University Hospital, Herlev Ringvej 75, Copenhagen, Denmark; 2 Department of Respiratory Medicine, Gentofte Hospital, Copenhagen University Hospital, Copenhagen, Denmark; 3 Respiratory and Allergy Research Group, Manchester Academic Health Science Centre, University Hospital South Manchester NHS Foundation Trust, Manchester, UK

**Keywords:** COPD, Heart failure, Spirometry

## Abstract

**Background:**

Chronic obstructive pulmonary disease (COPD) is an important differential diagnosis in heart failure (HF). However, routine use of spirometry in outpatient HF clinics is not implemented. The aim of the present study was to determine the prevalence of both airflow obstruction and non obstructive lung function impairment in patients with HF and to examine the effect of optimal medical treatment for HF on lung function parameters.

**Methods:**

Consecutive patients with HF (ejection fraction (EF) < 45%) and New York Heart Association (NYHA) functional class II-IV at 10 different outpatient heart failure clinics were examined with spirometry at their first visit and after optimal medical treatment for HF was achieved. airflow obstruction was classified and graded according to the GOLD 2011 revision.

**Results:**

Baseline spirometry was performed in 593 included patients and 71 (12%) had a clinical diagnosis of COPD. Mean age was 69 ± 11 years and mean EF was 30 ± 9%. Thirty-two % of the patients were active smokers and 53% were previous smokers. Mean FEV_1_ and FVC was 77.9 ± 1.7% and 85.4 ± 1.5% of predicted respectively. Obstructive pattern was observed in 233 (39%) of the patients. Of these, 53 patients (9%) had mild disease (GOLD I) and 180 (30%) patients had moderate to very severe disease (GOLD II-IV). No difference in spirometric variables was observed following up titration of medication.

**Conclusion:**

In stable patients with HF airflow obstruction is frequent and severely underdiagnosed. Spirometry should be considered in all patients with HF in order to improve diagnosis and treatment for concomitant pulmonary disease.

## Background

In patients with HF, chronic obstructive pulmonary disease (COPD) is an important differential or additional diagnosis due to sharing of risk factors (smoking) and symptoms (dyspnoea and fatigue). Diagnosing COPD in patients with HF is essential both because dyspnoea in patients with both diseases presents a particular challenge and because the combination of COPD and HF identifies a high-risk population [1–4]. Furthermore can a normal spirometric examination rule-out the coexistence of COPD and other lung disease which might avoid the use of unnecessary medication and help the clinician in focusing on the heart disease [5]. Studies of patients with HF have shown a prevalence of self-reported COPD of 10–33% [1, 3, 6] and studies of spirometry in patients with HF have shown an even higher prevalence of COPD (30–39%). However, most of these studies were either small [7, 8] or performed on patients with acute HF [9]. Recently, two larger studies suggest that spirometry might overestimate the presence of COPD in patients with heart failure and that the dynamic nature of lung function makes serial assessment mandatory [10, 11]. Previous studies of patients with HF have shown that acute and chronic congestion can lead to some reduction in lung function, and that treatment of congestion can partly reverse this [12, 13].

The purpose of the present study was to determine the prevalence of airflow obstruction in stable patients with HF and to examine the effect of optimal medical treatment for HF on lung function parameters.

## Methods

### Patients

All patients referred to 10 different Danish HF clinics for medical optimization between 1 January 2009 and 1 November 2011 were screened for inclusion in the study. Patients in the HF clinics were referred from general practitioners or other departments at the hospital. All patients with functional impairment corresponding to New York Heart Association (NYHA) functional class II-IV and with left ventricular ejection fraction (LVEF) < 45% were eligible for the study. Echocardiography was performed, by the referring departments or the hospital outpatient clinics, prior to inclusion in the study. Stable outpatients were examined with spirometry at the first visit to the HF clinic and again at the last visit where maximal possible doses of HF medication were achieved and patients were referred to further follow up by their general practitioner.

### Demographic data and medical history

Baseline demographic data, data on the use of tobacco, standard blood tests (creatinine, hemoglobin, sodium and potassium), previous medical history and the reason for development of HF were registered. At baseline and at the last visit doses and types of HF medication were registered. Doses of HF medications are in the following reported as % of doses recommended in the recent European guideline for betablockers and inhibitors of the renin angiotensine system [14].

### Spirometry

Forced expiratory volume in 1 s (FEV_1_) and forced vital capacity (FVC) were measured (Micro Medical MicroLab 3300) in a seated position without prior administration of a bronchodilator. At least three acceptable spirometric measurements were taken and the highest values were used. Measurements with a variation of FEV_1_ of up to 10% of the two highest recordings were accepted. Trained study nurses performed spirometry. Two investigators (KKI and MD) reviewed all the spirometry tracings and excluded those with signs of sub-optimal performance. The international recommendations were used to calculate predicted values [15] and in the following FEV_1_ and FVC are given as the percentages of these values. airflow obstruction was diagnosed according to GOLD criteria as an FEV_1_/FVC ratio < 0.7, and the degree of airflow impairment was graded according to the GOLD 2011 revision [16]. Mild airflow obstruction was defined as FEV_1_ ≥ 80% of predicted (GOLD I), moderate as FEV_1_ < 80% and ≥ 50% of predicted (GOLD II), severe as FEV_1_ < 50% and ≥ 30% of predicted (GOLD III) and very severe if FEV_1_ < 30% predicted (GOLD IV).

As a supplement to the GOLD criteria for defining airflow obstruction, the Lower Limit of Normality (LLN) criteria were used to analyse our data for comparative purposes. airflow obstruction is defined as having a FEV_1_/FVC ratio below the lower limit of normal, which is defined as the lower 5^th^ percentile of a healthy population as recommended by the American Thoracic Society and European Respiratory Community [17]. We used reference values from a large Danish study as baseline for the calculations of the FEV_1_/FVC ratio [18]. Patients with a spirometry with FEV1/FVC ≥ 0.7 and FVC < 80% of expected was defined as lung function impairment with a non-obstructive pattern/reserved ratio with impaired spirometry.

### Statistics

Comparisons between data in categories were performed using the chi-squared test, comparisons between continuous data using the Student’s *t*-test and comparisons between baseline and follow-up using the paired *t*-test. Multivariable analysis of variables associated with the presence of airflow obstruction was performed using logistic regression analysis with backward elimination (*p* = 0.1 as threshold for inclusion in the model).

Sensitivity, specificity, positive and negative predictive values were calculated with standard methods. All confidence intervals were constructed to have coverage of 95% and a *p*-value < 0.05 was considered significant. Statistical calculations were performed with SPSS software, version 20.0 (SPSS Inc., Chicago, IL, USA).

## Results

### Characterization of the patients

Screening was performed on 691 patients referred to the HF clinics. Of these 590 (85%) were included in the study. The main reasons for non-inclusion were patients inability to perform a spirometry of sufficient quality or patients not willing to participate in the study. Of the included patients 335 (57%) performed a follow-up spirometry, the main reason for missing follow-up spirometry was that the patients were terminated from follow-up earlier than expected from the HF outpatient clinic (due to death, severe comorbidity or the patient’s own decision). Twentyfour % of the patients were active smokers and 50% were previous smokers. Baseline characteristics for included and not included patients appear from Table 1. Patients not included were more often women, had lower NYHA-class and lower blood pressure than included patients. Apart from a slightly difference in sodium there were no difference between patients that did not complete follow-up and patients that completed the follow-up.

**Table 1:** Baseline characteristics for included and not included patients

	Included		*P*-value	Not included	*P*-value
	Baseline + FU performed (*n* = 338)	Only Baseline performed (*n* = 252)	FU/no FU	(*n* = 101)	Included/not included
Age years (SD)	69 (11)	68 (12)	0.61	71 (12)	0.59
Female gender n(%)	89 (27%)	67 (26%)	0.95	36 (36%)	0.04
NYHA III-IV n(%)	74 (25%)	61 (27%)	0.50	20 (20%)	0.03
Known COPD n(%)	44 (13%)	27 (11%)	0.27	12 (12%)	0.52
Previous or present smoking n(%)	238 (72%)	189 (78%)	0.14	73 (77%)	0.70
Body mass index kg/m^2^ (SD)	27 (5)	27 (5)	0.50	27 (6)	0.46
Systolic blood pressure mmHg (SD)	130 (22)	130 (25)	0.59	119 (22)	<0.01
Diastolic blood pressure mmHg (SD)	77 (13)	76 (11)	0.82	69 (15)	<0.01
Heart rate/minute (SD)	74 (15)	75 (14)	0.56	74 (13)	0.98
Ejection fraction % (SD)	31 (9)	31 (9)	0.46	31 (9)	0.52
eGFR’ ml/min (SD)	85 (124)	76 (102)	0.63	82 (118)	0.79
Sodium mmol/l (SD)	138 (4)	137 (4)	0.02	139 (1.4)	0.54
Potassium mmol/l (SD)	4.2 (0.5)	4.2 (0.5)	0.69	4.1 (0.4)	0.61
Treated with ACEI/ARB n(%)	308 (92%)	233 (92%)	0.89	87 (86%)	0.21
Treated with β-blocker n(%)	259 (77%)	197 (80%)	0.48	67 (66%)	0.31
Treated with loop-diuretics n(%)	200 (60%)	150 (61%)	0.88	53 (52%)	0.38
Treated with thiazides n(%)	29 (9%)	18 (7%)	0.26	10 (10%)	0.57
Treated with spironolactone n(%)	59 (18%)	53 (21%)	0.29	26 (26%)	0.06

‘estimated glomerular filtration rate

### Spirometric findings

**Fig. 1:**
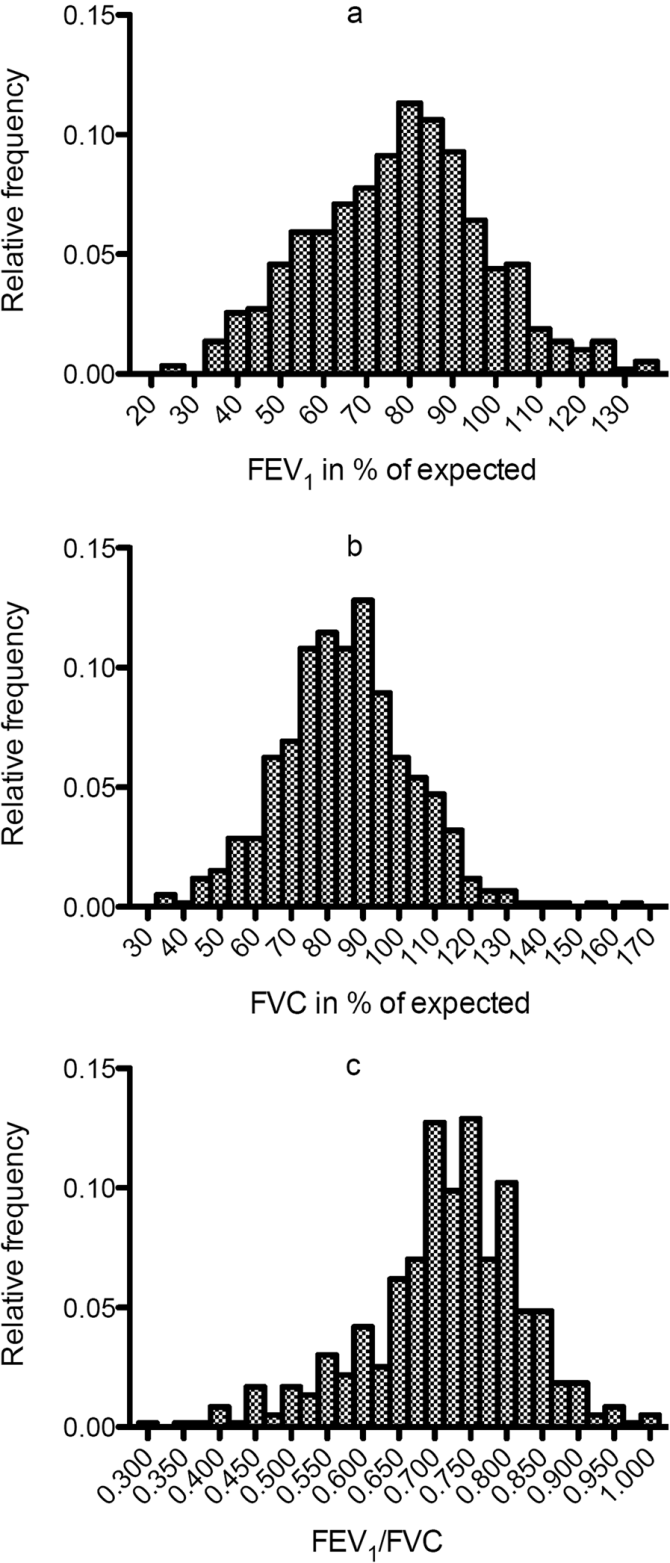
Histograms of spirometric variables (panel **a**; FEV_1_ in percentage of expected, panel **b**; FVC in percentage of expected, panel **c**; FEV_1_ /FVC)

At baseline mean FEV_1_ was 78% of expected (95% confidence interval 77–80%), mean FVC was 86% of expected (84–87%) and mean FEV_1_/FVC was 0.72 (0.71–0.72), data are presented in Fig. 1. Airflow obstruction (according to GOLD criteria) was present in 228 patients (38%) of which 51 (9%) were in GOLD grade of airflow obstruction I, 137 (23%) were in GOLD grade of airflow obstruction II, 38 (6%) were in GOLD grade of airflow obstruction III and 2 (0.3%) were in GOLD grade of airflow obstruction IV. A logistic regression was performed including all variables from Table 1. The only variables that independently predicted the presence of airflow obstruction were BMI, OR = 0.94 per increase in BMI of 1 kg/m2, (0.89–0.99), previous smoking, OR = 2.7 (1.0–7.3) and age 1.04 per year (1.01–1.08). Among present or previous smokers 43% of patients had an obstructive pattern while 24% of never smokers had an obstructive lung function impairment (*p* < 0.001). Furthermore were 34/35 (97%) of never smokers in GOLD grade of airflow obstruction 1 or 2 compared to 147/184 (80% of present or previous smokers (*p* < 0.001).

Furthermore, 130 (22%) had a lung function impairment with a non-obstructive pattern/ preserved ratio with impaired spirometry (i.e., FEV_1_/FVC ≥ 0.7 and FVC < 80% of expected), leaving 39% of the patients having a normal lung function.

### Association between spirometric findings and medical history

According to the patient records and the patients’ own information, 71 (12%) of the patients had previously been diagnosed with COPD (information of self-reported COPD was available in 582 (99%) of the patients). The association between airflow obstruction based on spirometry and self-reported COPD is presented in Table 2. Even though airflow obstruction based on spirometry was more frequent in patients with self-reported COPD (*p* < 0.01), 35% of the patients without known COPD had airflow obstruction. On the other hand 34% of the patients with self-reported COPD had no obstructive airflow obstruction (60% had normal spirometry and 40% had a restrictive pattern). Of the 228 patients with airflow obstruction only 47 (21%) had previously been diagnosed with COPD. The sensitivity of self-reported COPD compared to airflow obstruction based on spirometry was 0.21 (0.16–0.27), specificity was 0.93 (0.91–0.96), the positive predictive value was 0.66 (0.53–0.76) and the negative predictive value was 0.65 (0.61–0.69). Using logistic regression including variables from Table 1, BMI, OR = 0.94 per kg/m2, (0.89–0.99), previous smoking, OR = 2.7 (1.0–7.3) and age 1.04 per year (1.01–1.08) independently predicted the presence of obstructive lung function impairment.

**Table 2:** The association between COPD based on spirometry and self-reported COPD

	No obstructive airflow obstruction	Obstructive airflow obstruction			
		GOLD grade of airflow obstruction			
Self-reported COPD		I	II	III	IV
Absent n (%)	333 (65%)	50 (10%)	107 (21%)	20 (4%)	1 (0.2%)
Present n (%)	24 (34%)	1 (1%)	27 (38%)	18 (25%)	1 (1%)

### Comparison between LLN and GOLD criterias for airflow obstruction

Using LLN instead of GOLD criteria reduced the number of patients with airflow obstruction from 228 (38%) to 176 (30%) (*p* < 0.01). Comparing these criteria’s with self-reported COPD showed that 26% of the patients without known COPD had airflow obstruction and that 39% of the patients with known COPD did not have any airflow obstruction. The sensitivity of self-reported COPD compared to airflow obstruction based on spirometry using the LLN criteria was 0.24 (0.17–0.33), specificity was 0.93 (0.91–0.95), the positive predictive value was 0.61 (0.48–0.72) and the negative predictive value was 0.74 (0.70–0.78).

### Change in spirometry after medical uptitration in HF medication

**Fig. 2:**
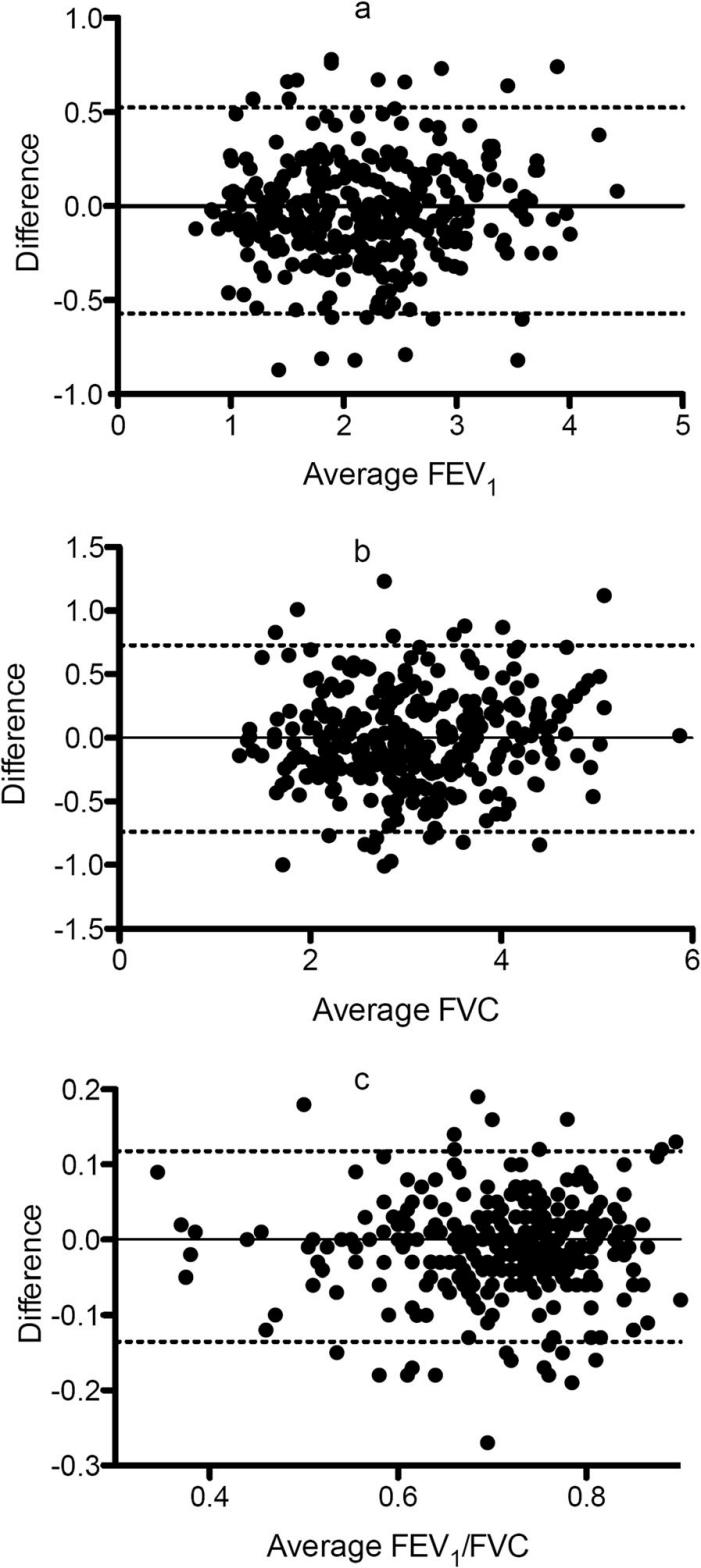
Bland-Altman plot of the difference in spirometric variables after and before up-titration of HF medication (panel **a**; FEV_1_, panel **b**; FVC, panel **c**; FEV_1_ /FVC)

Data on the 335 patients who completed the follow-up is presented in Table 3. Median time between baseline and follow-up was 174 days (inter quartile range 98–282). Blood pressure and heart rate were reduced, fewer patients were in NYHA class III and IV, and doses of ACE-inhibitors, beta-blockers and spironolactone increased during follow-up. In the entire population, mean values of FEV_1_, FVC and FEV_1_/FVC did not change significantly from baseline to follow-up. Values for FEV_1_ changed from 77.6% of expected to 76.4% of expected, *p* = 0.11; FVC from 84.9% of expected to 84.0% of expected, *p* = 0.27 and FEV_1_/FVC from 0.72 to 0.71, *p* = 0.22. In Fig. 2 Bland-Altman plots illustrate the change in FEV_1_, FVC and FEV_1_/FVC from follow-up to baseline as a function of the average values. It appears from the figure that lung function was stable over time and that any variability seems independent of level of lung function . Of the patients with airflow obstruction (FEV_1_/FVC < 0.7) at baseline 29 (12.7%) did not have airflow obstruction at follow-up, whereas 39 (10.8%) of the patients with no airflow obstruction at baseline had airflow obstruction at follow-up. Sensitivity of self-reported COPD compared to airflow obstructionbased on follow-up spirometry was 0.25 (0.18–0.33) and specificity was 0.95 (0.90–0.95), which not was different to results from the baseline spirometry (*p* > 0.05)

**Table 3:** Data on the 335 patients who completed the follow-up

	Baseline	Follow-up	*P*
NYHA III-IV n(%)	74 (22%)	33 (10%)	<0.01
Weight, kg (SD)	82 (19)	82 (20)	0.39
Systolic blood pressure, mmHg (SD)	129 (20)	124 (21)	<0.01
Diastolic blood pressure, mmHg (SD)	77 (14)	74 (11)	0.01
Heart rate, /minute (SD)	74 (15)	67 (13)	<0.01
ACE-inhibitor, % of recommended dose (SD)	46 (35)	68 (42)	<0.01
Beta-blocker, % of recommended dose (SD)	32 (30)	63 (38)	<0.01
Dose of furosemide, mg (SD)	61 (108)	56 (107)	0.03
Treated with spironolactone, n(%)	58 (18%)	73 (22%)	0.01
FEV_1,_ % of expected (SD)	77 (19)	76 (22)	0.11
FVC, % of expected (SD)	85 (18)	84 (21)	0.27
FEV_1_/FVC, (SD)	0.72 (0.10)	0.71 (0.11)	0.22

## Discussion

In a stable outpatient HF population more than one third of the patients had airflow obstruction and 78% of the patients had at least moderate to severe airflow obstruction (GOLD II-IV). Of these, only one in five had previously been diagnosed with COPD.. Even though the stable patients might have a degree of chronic congestion at baseline that is reduced after optimization of specific treatment for HF lung function parameters did not change. This suggests that congestion only plays a minor role in the observed lung function impairment. The prevalence of lung disease in patients with HF has previously primarily been described using self-reported COPD [1, 3, 6, 19]. The prevalence of COPD in these studies has varied between 10–33%. Only a handful of studies have examined patients with HF with spirometry. The findings are, however, relatively consistent, with approximately one third of the patients having airflow obstruction based on spirometry. One of the largest studies included 527 patients, but the study population consisted of patients admitted with acute HF, and congestion could therefore potentially have lead to overestimation of the lung function impairment [9]. Recently, two similar sized studies have shown that the airflow obstruction observed in patients with HF is dynamic and that serial measurements is mandatory to minimize the risk of false positive COPD diagnoses [10, 11]. The four minor studies were all in smaller and/or selected populations (118–187 patients) [7, 8, 20, 21]. None of the studies have performed serial spirometry and only one study used cut-off values for FEV_1_/FVC other than a fixed value of 0.7, which might overestimate the prevalence of airflow obstruction in an older population [7, 22].

The primary strength of the present study is that it is the largest study examining serial lung function in stable patients with HF. Furthermore, is this the only larger study that uses both LLN and GOLD criteria in the airflow obstruction diagnosis. Only 85% of the screening population was included in the study and follow-up was only possible in 57% of the included patients. However, only minor differences in baseline variables were observed between included and not included patients, which reduce the risk for selection bias. Furthermore follow-up was only possible in 57% of the patients. Nurses in the HF clinic performed the spirometry. Theses nurses had prior to the study only limited experience in performing spirometry. However, all nurses were prior to the study trained in spirometry and furthermore two experienced investigators (KI and MD) reviewed and approved all tracings prior to inclusion of the data in the study. The prevalence of undiagnosed asthma in this study is unknown and potentially some of the patients with airflow obstruction could have asthma and not COPD. A previous study of reversibility of airflow obstruction in patients with HF did not find any effect of an inhaled bronchodilator [9] and the risk of undiagnosed asthma in this rather old population is probably verylow. This is a screening study and the airflow obstruction diagnosis is solely based on spirometry, caution is therefore warranted when interpreting the prevalence of airflow obstruction in this study. The relatively high percentage of airflow obstruction in never smokers also underlines that an obstructive pattern not necessarily means that the patient has COPD in this study. Knowledge about smoking history is central in interpretation of a spirometry and the study would therefore have been strengthened by further knowledge of smoking history. Almost 13% of the patients with airflow obstruction at baseline did not have airflow obstruction at follow-up. These patients had only minor airflow obstruction and the changes were below 10%. The explanations for this finding might be several and include that some patients had asthma, some had minor congestion, and maybe most likely biological and examiner variability.

Over-diagnosis of COPD might be as important a clinical issue as under-diagnosis. This is partly due a potential overuse of medication for COPD and partly due to the possibility that clinicians think that dyspnoe is due to lung disease instead of heart disease. The use of the LLN criteria instead of a fixed ratio has been suggested to reduce the problem with over-diagnosis. Previous studies have shown that the number of patients with airway obstruction is reduced with approximately one third if LLN is used instead of a fixed ratio [23]. A small study has examined this issue in a population of heart failure patients (*n* = 89) [24]. They found a reduction of nearly 50% of the number of patients with air way obstruction if LLN is used. In the present study we found a significant, but minor than previously reported, difference between LLN and fixed ratio diagnoses of airway obstruction. This finding suggests that over-diagnosis of COPD in HF patients with the use of criteria based on a fixed ratio might be less than previously thought. On the other hand, the minor differences between earlier studies and this study might at least in part be due to difference in age of patients included in the studies as the difference between LLN and fixed ratio for diagnosing airflow obstruction is age dependent. The reason for the high coexistence of airflow obstruction and HF is probably multifactorial. The sharing of the common risk factor - smoking - is one important factor. However, it has recently been proposed that low-grade inflammation observed in COPD could be a link between the two diseases [25–29]. The role of congestion on pulmonary function has been debated and results from previous studies are conflicting. Acute saline infusion in patients with HF has been shown to increase airflow obstruction but not to induce restriction [30]. Similarly, studies in patients with mitral valve stenosis have shown that bronchial hyperreactivity was associated with increased filling pressures of the left ventricle [12]. In contrast, other studies have shown that FVC and not FEV_1_ were related to degree of congestion in heart failure patients [13, 31], and that heart transplantation normalized the restriction in the majority of patients [31]. Still, most studies show that both FEV_1_ and FVC are correlated to congestion and that medical treatment or surgery that alleviates congestion improves both parameters [32–36]. In the present study, optimal treatment of HF changed neither FEV_1_ nor FVC. This could at least partly be explained by patients already being stable at baseline and that the level of congestion therefore probably was minimal. The lack of change in body weight from baseline to follow-up supports this notion.

In this study we found that almost one fourth of the patients had a lung function impairment with a non-obstructive pattern. Treatment of this condition can be difficult and therefore might the clinical relevance of this finding be limited. While some of the patients might have an impaired lung function du to non-pulmonary causes (adipositas or chronic congestion) some of the patients could potentially have severe primary lung disease (i.e., pulmonary fibrosis). The relevance of this finding should therefore be investigated in future studies.

We could confirm previous findings of a very low agreement rate between self-reported COPD and airflow obstruction diagnosed by spirometry. The sharing of symptoms –dyspnoea - implicates that there are significant diagnostic challenges between HF and COPD. Both auscultation [37] and history [23] is difficult to use for differentiation between heart disease and lung disease and even though natriuretic peptides can diagnose HF, they can not exclude pulmonary disease. Diagnosing and treating undiagnosed COPD might be essential in patients with HF as a growing level of evidence has shown that targeted treatment for COPD reduces symptoms, improves quality of life and may improve prognosis [38]. Furthermore, the knowledge of patients having COPD may reduce the risk of overdosing diuretics due to misinterpretation of the background for patients having dyspnoea. It therefore seems relevant to perform spirometry – a simple, cost-effective, non-invasive and objective examination – in all patients with HF.

## Conclusion

In stable patients with HF airflow obstruction is frequent and severely underdiagnosed. Spirometry should be considered in all patients with HF in order to improve diagnosis and treatment for concomitant pulmonary disease.

## Abbreviations

COPD: Chronic obstructive pulmonary disease
EF: Ejection fraction
FEV_1_: Forced expiratory volume in 1 second
FVC: Forced vital Capacity
GOLD: Global initiative for chronic obstructive lung disease
HF: Heart failure
LLN: Lower limit of normality
NYHA: New York Heart Association
